# Tissue-Specific Oxidative Stress Modulation by Exercise: A Comparison between MICT and HIIT in an Obese Rat Model

**DOI:** 10.1155/2019/1965364

**Published:** 2019-07-14

**Authors:** Carole Groussard, Florie Maillard, Emilie Vazeille, Nicolas Barnich, Pascal Sirvent, Yolanda F. Otero, Lydie Combaret, Elise Madeuf, Antoine Sourdrille, Geoffroy Delcros, Monique Etienne, Allison Teixeira, Pierre Sauvanet, Vincent Pialoux, Nathalie Boisseau

**Affiliations:** ^1^Univ-Rennes, Laboratoire M2S-EA 7470, F-35000 Rennes, France; ^2^Université Clermont Auvergne, Laboratoire AME2P, EA 3533, Clermont-Ferrand, France; ^3^Université Clermont Auvergne, M2iSH, UMR 1071 INSERM, UCS INRA 2018, Clermont-Ferrand, France; ^4^Université Clermont Auvergne, CHU Clermont-Ferrand, Service des Maladies de L'appareil Digestif, Clermont-Ferrand, France; ^5^INRA, Unité de Nutrition Humaine (UNH, UMR 1019), Clermont-Ferrand, Université Clermont Auvergne, France; ^6^Univ Lyon, Université Lyon 1, LIBM EA 742, Villeurbanne, France; ^7^Institut Universitaire de France, Paris, France; ^8^CRNH Auvergne, Clermont-Ferrand, France

## Abstract

**Background and Aim:**

Exercise is an effective strategy to reduce obesity-induced oxidative stress. The purpose of this study was to compare the effects of two training modalities (moderate-intensity continuous training (MICT) and high-intensity interval training (HIIT)) on the pro/antioxidant status of different tissues in obese Zucker rats.

**Methods:**

Eight-week-old male Zucker rats (*fa*/*fa*, *n* = 36) were subdivided in three groups: MICT, HIIT, and control (no exercise) groups. Trained animals ran on a treadmill (0° slope), 5 days/week for 10 weeks (MICT: 51 min at 12 m·min^−1^; HIIT: 6 sets of 3 min at 10 m·min^−1^ followed by 4 min at 18 m·min^−1^). Epididymal (visceral) and subcutaneous adipose tissue, gastrocnemius muscle, and plasma samples were collected to measure oxidative stress markers (advanced oxidation protein products (AOPP), oxidized low-density lipoprotein (oxLDL)), antioxidant system markers (ferric-reducing ability of plasma (FRAP), superoxide dismutase (SOD), catalase, and glutathione peroxidase (GPx) activities), and prooxidant enzymes (NADPH oxidase and xanthine oxidase (XO) activities, myeloperoxidase content).

**Results:**

Compared with the control, MICT increased GPx and catalase activities and the FRAP level in epididymal adipose tissue. HIIT increased the AOPP level in subcutaneous adipose tissue. In the muscle, HIIT increased both SOD and GPx activities and reduced the AOPP level, whereas MICT increased only SOD activity. Finally, plasma myeloperoxidase content was similarly decreased by both training modalities, whereas oxLDL was reduced only in the MICT group.

**Conclusion:**

Both HIIT and MICT improved the pro/antioxidant status. However, HIIT was more efficient than MICT in the skeletal muscle, whereas MICT was more efficient in epididymal adipose tissue. This suggests that oxidative stress responses to HIIT and MICT are tissue-specific. This could result in ROS generation via different pathways in these tissues. From a practical point of view, the two training modalities should be combined to obtain a global response in people with obesity.

## 1. Introduction

In 2016, there were approximately 650 million people with obesity worldwide [1], making obesity a major public health problem, mainly caused by increased intake of energy-dense food and physically inactive lifestyles [2, 3]. In this context, physical activity appears as an effective strategy for obesity prevention and management [4].

For years, moderate-intensity continuous training (MICT) has been the most popular exercise modality for improving body composition, cardiorespiratory fitness, insulin resistance, and lipid profile [2, 5]. However, the long-term adherence to this type of training is low, and many people stop exercising mainly because of lack of time and/or loss of motivation and gratification [6]. Recent studies have demonstrated that high-intensity interval training (HIIT), which consists in alternating short periods of high-intensity exercise with periods of light exercise (recovery) [7], is perceived as less monotonous and more enjoyable [8], thus significantly increasing participation and adherence [9, 10]. Although not all studies are unanimous [11–13], most agree that this type of training is also a time-effective alternative to MICT, leading to higher weight loss, particularly visceral fat mass, and better improvement of the metabolic profile and cardiorespiratory fitness in people with obesity [14–16].

In 2004, Furukawa et al. were the first to demonstrate in obese mice and in humans with obesity that fat accumulation is positively associated with systemic oxidative stress, suggesting that the oxidative stress increase in people with obesity could be explained by reactive oxygen species (ROS) overproduction in adipose tissue [17]. In addition, ectopic fat accumulation in the muscle promotes ROS production in this tissue [18]. MICT can reduce oxidative stress by increasing antioxidant enzyme activity and decreasing ROS production in the skeletal muscle [19], adipose tissue [20], and vascular tissue [21]. MICT also reduces systemic oxidative stress, particularly in people with obesity [22–24].

As fat mass accumulation increases oxidative stress [17] and HIIT leads to greater fat mass loss, particularly visceral fat [15], we hypothesized that this training modality could have a greater effects on the pro/antioxidant status compared with MICT in a rat model of obesity (Zucker rats). To test this hypothesis, we compared the effects of 10 weeks of HIIT and MICT on the pro/antioxidant status (antioxidant system, prooxidant enzymes, and oxidative stress markers) in different tissues (epididymal and subcutaneous adipose tissues, muscle, and plasma) in male Zucker rats.

## 2. Materials and Methods

### 2.1. Ethical Approval

The experimental protocol was approved by the local ethics committee (CE2A-02, Auvergne, France—protocol number 3075-2015120813375547) and was in accordance with the current legislation on animal experimentation (*Guide for the Care and Use of Laboratory Animals, Eighth Edition 2011*). Moreover, the experiments were carried out according to the local institution's animal welfare committee.

### 2.2. Animal Model and Experimental Groups

Seventy-five adult (8-week-old) male Zucker *(fa/fa)* rats were purchased from Charles River and were housed individually in an air-conditioned room with controlled temperature (21°C) and a reverse 12/12 h light/dark cycle. Tap water and food (3% lipids, 16% proteins, 60% carbohydrates, 5% minerals, and 4% fibers—SAFE A04, France) were available ad libitum.

After 10 days of acclimatization (including 5 days of treadmill exercise), 36 rats were selected for the study after a running test. During running habituation, rats that refused to run spontaneously after the treadmill started were excluded from the study. The runners were then randomly divided into the control group (*n* = 12), the MICT group (*n* = 12), and the HIIT group (*n* = 12). Animals in the three groups had similar weight, body fat, and fasting glucose level.

### 2.3. Experimental Design

Body weight was measured weekly in all animals, and the amount of food consumed was registered daily. Body composition was measured by MRI (Echo Medical Systems, Houston, TX), and epididymal fat pads were weighted *post-mortem*. At the end of the protocol (week 10) and 48 h after the last exercise session, the animals were killed by dislocation before tissue harvesting. Whole blood samples were immediately centrifuged at 2000 × g at 4°C for 10 min to obtain plasma. Aliquots were stored at -80°C until analysis. Muscles (gastrocnemius) and subcutaneous/epididymal adipose tissue samples were collected, weighed, and immediately frozen in liquid nitrogen and stored at -80°C until analysis.

### 2.4. Training Protocol

**Figure 1:**
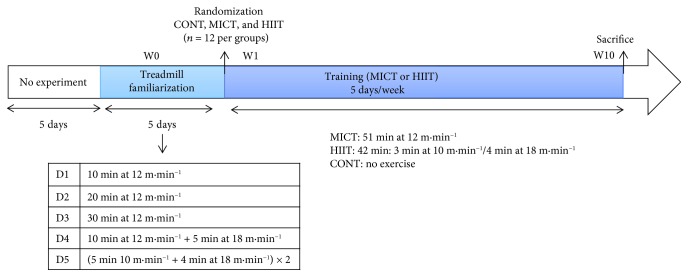
Experimental design. CONT: control (no exercise); MICT: moderate-intensity continuous training; HIIT: high-intensity interval training; W: week.

One week before the experimental period, the animals were familiarized with the training procedures using a low-intensity running protocol, as described in Figure 1. The treadmill habituation protocol finished three days before the beginning of the experimental period to avoid acute interference with the baseline measurements.

For the training protocol (10 weeks), the animals ran on a treadmill especially designed for rats (Panlab, Harvard Apparatus, LE 8710R), and all sessions were performed during the dark cycle (active period). Before each training session, animals in the MICT and HIIT groups performed a regular warm-up exercise at 10 m·min^−1^ for 5 min. The MICT group ran for 51 min at 12 m/min, 5 times per week for 10 weeks. The HIIT group alternated 3 min at 10 m/min and 4 min at 18 m/min (6 sets; 5 times per week for 10 weeks). The protocols were originally designed to have identical total running distance between groups, as proposed by [25–27]. Animals in the control group were managed identically as those in the MICT and HIIT groups but without exercise. Control rats were placed in the same room during the training sessions to account for the potential stress induced by environment changes.

### 2.5. Biochemical Analyses

Muscle and adipose tissue samples were ground in liquid nitrogen, homogenized (10%, *w*/*v*) in 1x PBS/0.5 mM EDTA on ice, and centrifuged at 12000 × g at 4°C for 10 min. Homogenates were stored in aliquots at -80°C. Total protein concentration was determined using the BCA Protein Assay Kit (Sigma-Aldrich, St Louis, USA) following the manufacturer's instructions. All the products used for oxidative stress marker measurements were from Sigma-Aldrich, and spectrophotometric measurements were performed on a TECAN Infinite 2000 plate reader (Männedorf, Switzerland). Results obtained with the skeletal muscle and adipose tissue samples were normalized to the total protein content to account for body weight variations during the experiment. Measurements were done in triplicates.

#### 2.5.1. Oxidative Stress Markers

AOPP were determined according to the method by Witko-Sarsat et al. using a spectrophotometer and calibrated with a chloramine-T solution that absorbs at 340 nm in the presence of potassium iodide [28]. The absorbance of the reaction mixture was read at 340 nm. AOPP concentrations were expressed as *μ*mol·L^−1^ of chloramine-T equivalents. The intra-assay coefficient of variation (CV) was 5.4%.

oxLDL concentration was measured in plasma using an ELISA kit (ELISA KIT, Elabscience®) according to the manufacturers' recommendations. Absorbance was read at 450 nm.

#### 2.5.2. Antioxidant System Markers

Plasma SOD activity was determined using the method by Oberley and Spitz [29] based on the degree of SOD inhibition of the reaction between superoxide radicals, produced upon hypoxanthine oxidation by xanthine oxidase, and nitroblue tetrazolium (NTB). The blue formazan product subsequently formed was read at 560 nm for 5 min. The intra-assay CV was 5.6%.

Plasma GPx activity was determined using a modified version of the method described by Paglia and Valentine [30]. GPx activity is represented by the rate of NADPH oxidation to NADP+ after addition of glutathione reductase, reduced glutathione, and NADPH using H_2_O_2_ as the substrate. NADPH extinction was read at 340 nm for 5 min [30]. The intra-assay CV was 4.6%.

Plasma catalase activity was determined using the method described by Johansson and Borg [31] with H_2_O_2_ as the substrate and formaldehyde as the standard. Catalase activity was determined by monitoring the formaldehyde formation rate (read at 540 nm for 20 min) induced by the reaction of methanol and H_2_O_2_ using catalase as the enzyme [31]. The intra-assay CV was 3.1%.

Plasma FRAP was determined by spectrophotometry using the manual method described by Benzie and Strain [32]. FRAP concentration was calculated using an aqueous solution of a known Fe^2+^ concentration (FeSO_4_-7H_2_O) as the standard. Each sample was mixed at 37°C with a FRAP working solution that contains buffer acetate, 2,4,6-Tris(2-pyridyl)-s-triazine (TPTZ), and ferric chloride (FeCl_3_-6H_2_O). The Fe^2+^-TPTZ complex formed was read at 593 nm after 4 min. The intra-assay CV was 2.9%.

#### 2.5.3. Prooxidant Enzymes

Nicotinamide adenine dinucleotide phosphate (NADPH) oxidase (NOX) and xanthine oxidase (XO) activities were determined in plasma, as previously described [33] by the reaction of NTB with superoxide produced by hypoxanthine or NADPH with XO and NOX, respectively. NOX and XO activities were calculated by measuring spectrophotometrically the kinetic appearance of the complex formed by superoxide and NTB at 560 nm for 10 min.

Myeloperoxidase concentration was evaluated in plasma samples using a commercial ELISA kit (Myeloperoxidase DuoSet ELISA, R&D Systems, Minneapolis, MN, USA). The optical density was determined at 450 nm. The intra-assay CV was 2.9%.

### 2.6. Statistical Analysis

Results are expressed as the mean ± standard deviation (SD). Normality was checked using Kolmogorov-Smirnov's test. The assumption of homogeneity of variance was assessed using the Bartlett *F*-test. When the conditions of normality and homogeneity of variance were respected, one-way (to analyse data between the groups) or two-way mixed model ANOVAs with repeated measures (group, time, and group×time interaction) were run and a Newman-Keuls post hoc test was applied when the ANOVA reached significance level (*p* < 0.05). All data were analysed using the Statistica software.

## 3. Results

### 3.1. Animals' Characteristics

Measurement of whole-body mass, total fat mass (FM: g and %) and fat-free mass (FFM: g) in the three groups is presented in Table 1. The evolution of whole-body mass and FFM was comparable among groups during the study period (10 weeks). However, FM (g and %) was lower in the MICT and HIIT groups than in the CONT group at week 5 (*p* < 0.05) and in the HIIT group than in the CONT and MICT groups at the end of the protocol (*p* < 0.05).

Finally, cumulated food intake did not differ between groups (1699.33 ± 236.20 g for the CONT, 1852.91 ± 137.24 g for the MICT group, and 1698.33 ± 140.34 g for the HIIT group; *p* > 0.05).

These data are included in another article dedicated to the effects of HIIT and MICT on gut-adipose tissue cross-talk in obese Zucker rats [34].

### 3.2. MICT and HIIT Effects on Muscle Pro/Antioxidant Status

**Figure 2:**
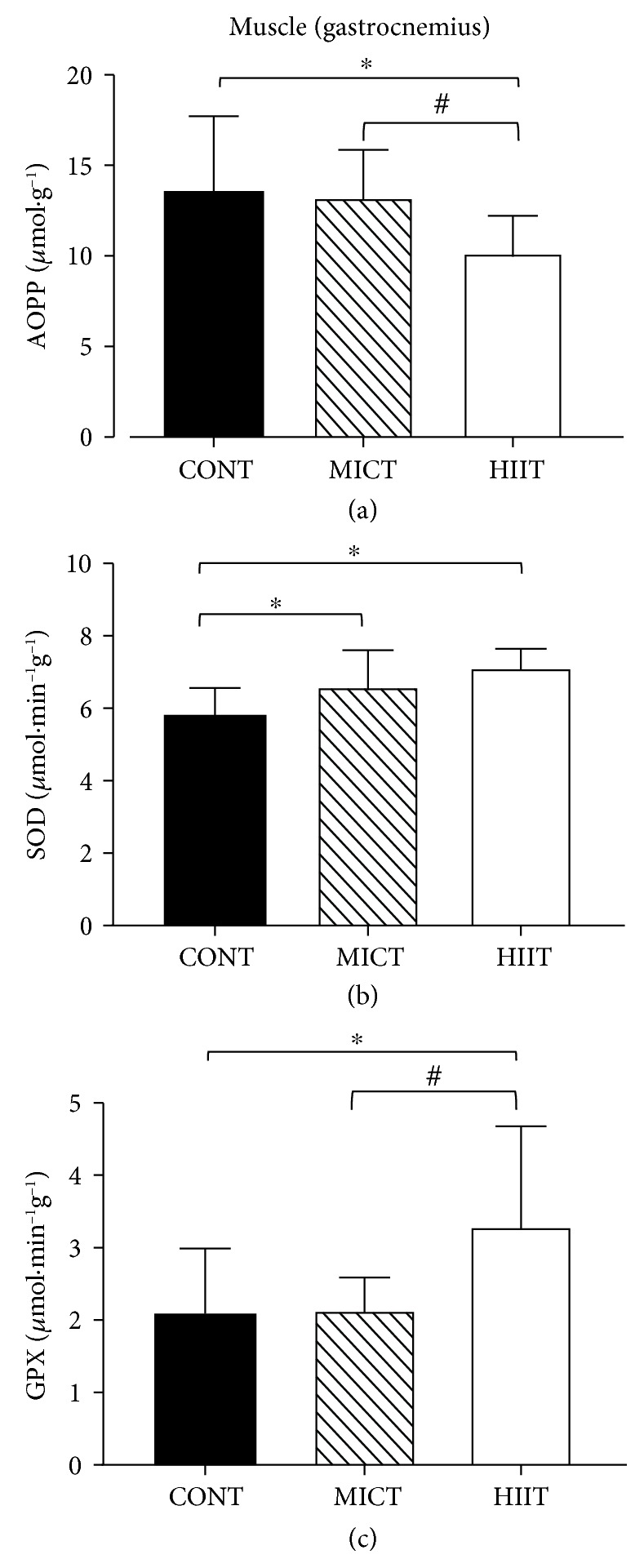
Effects of 10 weeks of exercise on (a) AOPP level (*μ*mol·g^−1^), (b) SOD (*μ*mol·min^−1^g^−1^), and (c) GPx activities (*μ*mol·min^−1^g^−1^) in gastrocnemius samples from the three groups. Data are the mean ± SD. ^∗^*p* < 0.05, compared with CONT; ^#^*p* < 0.05, compared with MICT. CONT: control (no exercise); MICT: moderate-intensity continuous training; HIIT: high-intensity interval training; AOPP: advanced oxidation protein products; SOD: superoxide dismutase; GPx: glutathione peroxidase.

Analysis of the pro/antioxidant status in gastrocnemius samples from the three groups at the end of the study (week 10) showed that HIIT reduced significantly the level of advanced oxidation protein products (AOPP) compared with the control (10.17 ± 2.05 *μ*mol · g^−1^ and 13.59 ± 4.14 *μ*mol · g^−1^, respectively; *p* < 0.05) and also with MICT (13.24 ± 2.61 *μ*mol · g^−1^; *p* < 0.05) (Figure 2(a)).

Superoxide dismutase (SOD) activity was significantly and similarly increased by both training modalities (7.13 ± 0.51 and 6.61 ± 1.00 *μ*mol · min^−1^g^−1^ for HIIT and MICT, respectively) compared with the control (5.82 ± 0.74 *μ*mol · min^−1^g^−1^) (Figure 2(b)).

Glutathione peroxidase (GPx) activity was significantly higher in the HIIT (3.29 ± 1.38 *μ*mol · min^−1^g^−1^) than in the MICT (2.14 ± 0.45 *μ*mol·min^−1^g^−1^) and control groups (2.09 ± 0.90 *μ*mol · min^−1^g^−1^) (*p* < 0.05) (Figure 2(c)).

No training effect was observed for the other pro/antioxidant status markers (Table 2).

### 3.3. MICT and HIIT Effects on the Adipose Tissue Pro/Antioxidant Status

**Figure 3:**
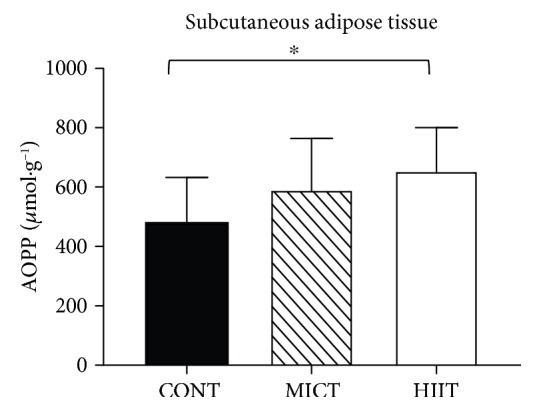
Effects of 10 weeks of exercise on the AOPP level (*μ*mol·g^−1^) in subcutaneous adipose tissue samples from the three groups. Data are the mean ± SD. ^∗^*p* < 0.05, compared with the CONT group. CONT: control (no exercise); MICT: moderate-intensity continuous training; HIIT: high-intensity interval training; AOPP: advanced oxidation protein products.

At the end of the study (week 10), the subcutaneous adipose tissue pro/antioxidant status was comparable in the three groups (Table 3), except for AOPP that was significantly higher in the HIIT than in the control group (655.50 ± 145.29 *μ*mol · g^−1^ vs. 482.40 ± 150.27 *μ*mol · g^−1^, respectively; *p* < 0.05) (Figure 3).

**Figure 4:**
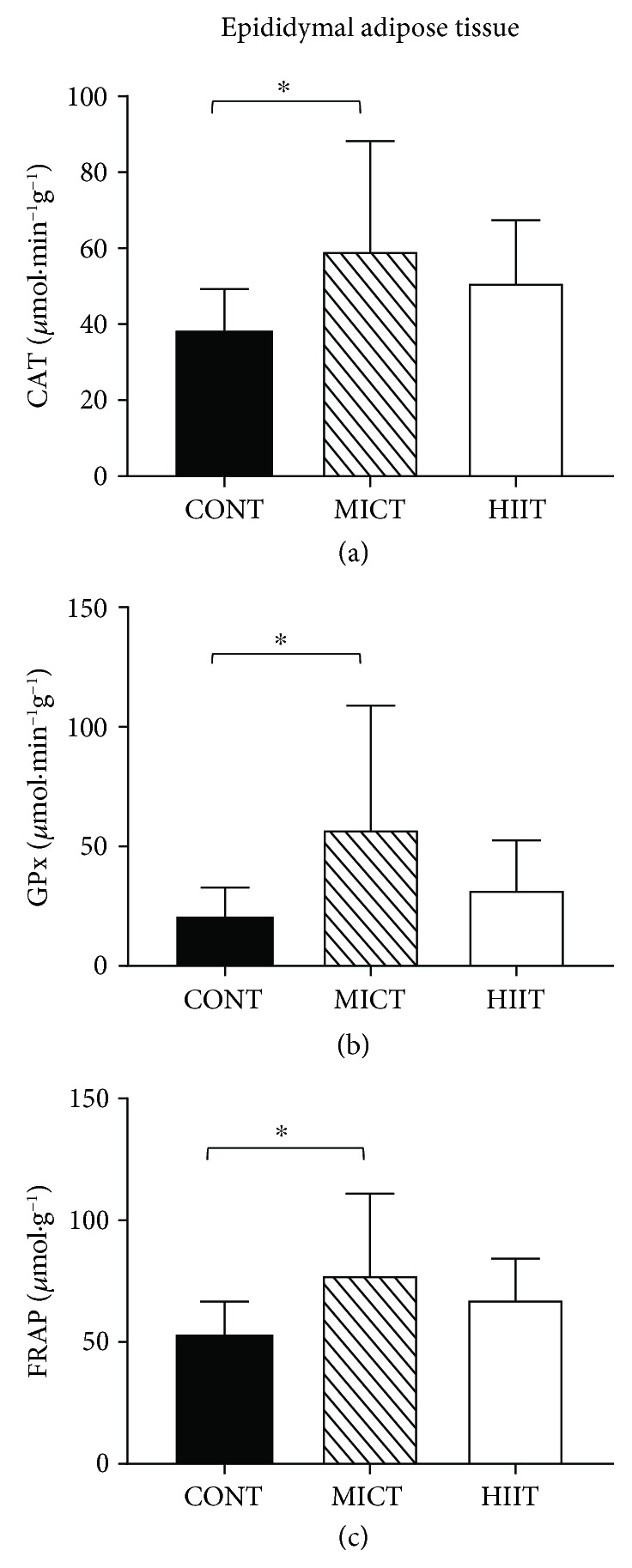
Effects of 10 weeks of exercise on (a) CAT (*μ*mol·min^−1^g^−1^) and (b) GPx (*μ*mol·min^−1^g^−1^) activities, and (c) FRAP (*μ*mol·g^−1^) in the epididymal adipose tissue. Data are mean ± SD. ^∗^*p* < 0.05, compared with the CONT group. CONT: control (no exercise); MICT: moderate-intensity continuous training; HIIT: high-intensity interval training; CAT: catalase; GPx, glutathione peroxidase; FRAP: ferric-reducing antioxidant power.

In epididymal (visceral) adipose tissue, only MICT significantly increased the activity of catalase (59.47 ± 28.74 *μ*mol · min^−1^g^−1^ in the MICT group and 38.31 ± 11.01 *μ*mol · min^−1^g^−1^ in the control group; *p* < 0.05). The activity of GPx (57.31 ± 51.62 *μ*mol · min^−1^g^−1^ in the MICT and 20.52 ± 12.24 *μ*mol · min^−1^g^−1^ in the control group; *p* < 0.05) and ferric-reducing ability of plasma (FRAP) (77.79 ± 33.17 in the MICT group and 53.08 ± 13.64 *μ*mol · g^−1^ in the control group; *p* < 0.05) is also increased only by MICT (Figure 4).

No training effect was observed for the other tested markers (Table 3).

### 3.4. MICT and HIIT Effects on the Systemic Pro/Antioxidant Status

**Figure 5:**
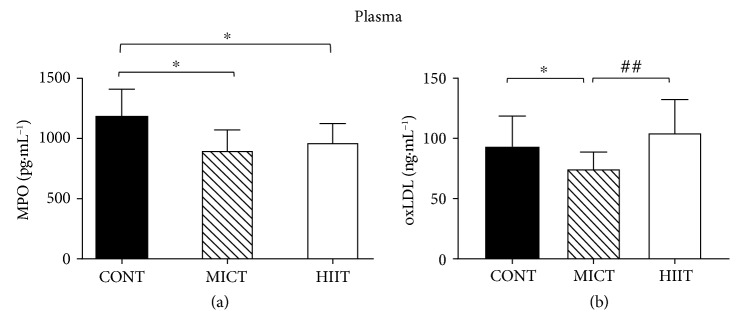
Effects of 10 weeks of exercise on (a) MPO content (pg·mL^−1^) and (b) oxLDL (ng·mL^−1^) level in plasma. Data are the mean ± SD. ^∗^*p* < 0.05, compared with CONT. ^##^*p* < 0.01, compared with MICT. CONT: control (no exercise); MICT: moderate-intensity continuous training; HIIT: high-intensity interval training; MPO: myeloperoxidase; oxLDL: oxidized low-density lipoprotein.

At week 10, myeloperoxidase content was similarly reduced by both training modalities (975.03 ± 158.02 for the HIIT group and 909.12 ± 170.84 pg · mL^−1^ for the MICT group) compared with the control (1196.63 ± 224.33 pg · mL^−1^) (*p* < 0.05) (Figure 5(a)).

However, only MICT significantly reduced oxidized low-density lipoprotein (oxLDL) level in plasma compared with the control (74.93 ± 13.82 ng · mL^−1^ and 93.12 ± 25.49 ng · mL^−1^, respectively; *p* < 0.05) and also with HIIT (74.93 ± 3.99 ng · mL^−1^ and 104.82 ± 27.44 ng · mL^−1^, respectively; *p* < 0.05) (Figure 5(b)).

HIIT and MICT did not modulate plasma SOD activity (13.14 ± 3.95 for the HIIT group and 13.71 ± 4.47 *μ*mol · min · L^−1^ for the MICT group) compared with the control (12.65 ± 4.15 *μ*mol · min · L^−1^).

## 4. Discussion

This study is the first to compare the effects of two exercise training modalities (MICT vs. HIIT) on the pro/antioxidant status in obese rats focusing especially on three main tissues: (1) adipose tissue which is the main tissue involved in ROS production in the case of obesity, (2) the muscle which is involved in exercise and where ROS are mainly produced, and (3) the blood which reflects the systemic aspect. We hypothesized that HIIT, by decreasing total and visceral fat mass to a greater extent than MICT, would lead to greater benefits on the pro/antioxidant status. As expected, HIIT led to higher reduction of the FM percentage than MICT (*p* < 0.05) at the end of the protocol; however, our results indicate that HIIT improved the pro/antioxidant status more efficiently than MICT only in the muscle, where ROS are mainly produced during exercise. Conversely, MICT was more efficient in epididymal adipose tissue.

In this study, we analyzed the effects of MICT and HIIT in Zucker rats (*fa/fa*). This animal model of obesity is the best known and most widely used model of genetic obesity and displays the whole biochemical and metabolic pathological outcomes of the disease (hyperinsulinemia, hyperlipidaemia and insulin resistance, and adipocyte hypertrophy/hyperplasia) [35, 36]. Zucker rats are significantly hyperphagic and quickly develop obesity (at around 4 weeks of age) due to a mutation in the leptin receptor (fa gene), like in human obesity [37]. Moreover, obese Zucker rats are a good model to test the beneficial effects of therapeutic intervention programs based on physical activity [38, 39].

We focused our attention on the pro/antioxidant status because oxidative stress has been proposed to be the unifying mechanism in the development of major obesity-related comorbidities, particularly cardiovascular disease and diabetes [40]. Several mechanisms may explain the oxidative stress increase in people with overweight/obesity, particularly (i) a decrease of antioxidant defenses, (ii) an increase in mitochondrial ROS production due to excessive energy expenditure, (iii) an increase in plasma lipids that are oxidation targets, (iv) an increase of the leptin level that may stimulate intracellular ROS production, and (v) ROS overproduction by adipose tissue due to cytokine release [41]. In this context, interventions that can protect against oxidative stress are essential to prevent obesity-related complications and should be encouraged [42]. In addition, physical training is likely to be the only known method that reduces oxidative stress independently of body adiposity [42]. In our study, we demonstrated that each training modality induced tissue-specific modulations of oxidative stress.

Our results on the differential adipose tissue pro/antioxidant status modulation by the two training modalities bring new knowledge to the existing literature. In epididymal adipose tissue, only MICT induced beneficial effects by increasing GPx and catalase activities and the FRAP level. Conversely and surprisingly, in subcutaneous adipose tissue, HIIT increased the AOPP level. It is known that basal oxidative stress increases in the white adipose tissue of obese animals [17, 43, 44] due to upregulation of NADPH oxidase subunits and downregulation of antioxidant enzymes (SOD and GPx), leading to higher H_2_O_2_ production and lipid peroxidation [17]. Very few studies investigated the adipose tissue pro/antioxidant status in response to training, and to our knowledge, no study compared HIIT and MICT in obese animals. Like others [45, 46], we found that the activity of antioxidant enzymes (catalase and GPx) was increased in epididymal adipose tissue after MICT, confirming that exercise training acts as an antioxidant also in this tissue. Conversely, oxidative stress markers were not decreased in adipose tissue after MICT. This is different from a previous study [47] showing that after 10 weeks of aerobic training, the protein carbonyl group level was reduced in epididymal fat depots. This discrepancy could be explained by the different exercise intensity of the training protocols (20 m·min^−1^ vs. 12 m·min^−1^ in our study).

In our study, MICT specifically modulated the pro/antioxidant status in visceral adipose tissue, but not in subcutaneous adipose tissue, as previously reported by Sakurai et al. [20]. These authors showed that exercise training increases manganese-dependent SOD (Mn-SOD) activity and decreases lipid peroxidation in epididymal adipose tissue, but not in subcutaneous adipose tissue [20]. The underlying mechanisms remain to be elucidated. The sensitivity of adipocytes to exercise-induced changes could play a role because the catecholamine-induced lipolytic response is higher in omental (visceral adipose tissue) than in subcutaneous adipocytes [48]. Finally, the absence of HIIT protective effect in the two adipose tissues suggests that MICT and HIIT act on different redox mechanisms in function of the tissue.

In the muscle, both training modalities increased SOD activity; however, only HIIT promoted significantly GPx activity and decreased AOPP, suggesting that in obese rats, the muscle pro/antioxidant status is improved more efficiently by HIIT than by MICT. The significant AOPP reduction in the HIIT group might be explained by the induction of GPx activity observed with this training modality. Therefore, it is likely that by improving the muscle antioxidant defenses, particularly GPx activity, HIIT reduces the muscle oxidative damage. In normal-weight rats, high-intensity training, but not continuous training, increases GPx activity in the muscle [49]. The underlying mechanisms are not fully understood, but HIIT could induce the activation of redox-sensitive protein signalling pathways [50] via higher ROS production in the muscle. Indeed, as muscle ROS production is related to the exercise intensity [51], during HIIT, each bout of intense exercise might induce an important increase in H_2_O_2_ production, thus outmatching the muscle antioxidant defenses. Conversely, during MICT, the hydroxide peroxide (H_2_O_2_) content can be more easily regulated. This hypothesis is supported by the finding that compared with continuous exercise, intermittent exercise induces a greater activation of redox-dependent signalling pathways due to the higher metabolic demand [52]. Another hypothesis can be formulated to explain the greater increase in antioxidant enzyme activities in the muscle in response to HIIT. We cannot exclude decrease in ROS production by mitochondria as previously demonstrated in healthy people, in response to aerobic training [53] and/or a decrease in ROS production due to a greater loss of intramuscular triglycerides (IMTG) in response to HIIT. Indeed, although in healthy people IMTG is used as an energy source and may be increased by endurance training exercise [54, 55], Ko et al. [56] demonstrated in an obese mouse model that 8 weeks of treadmill exercise contributes to decreased IMTG volume by activating lipolysis factors. To our knowledge, no study investigated the effect of different modalities of training (HIIT vs. MICT) on intramuscular triglycerides and especially in relation to the oxidative stress status. It may be hypothesized that HIIT decreases IMTG more than MICT because of the higher IMGT lipolysis resulting from higher lactate production. In turn, ROS production (which is related to this percentage in obese subjects) should decrease [57].

Concerning plasma markers of the pro/antioxidant status, both training modalities similarly reduced the myeloperoxidase level, but only MICT could decrease the oxLDL level. The decrease of myeloperoxidase, a marker of neutrophil activation/degranulation and superoxide radical production [58], by both training modalities is relevant because recent studies suggest that elevated circulating myeloperoxidase levels could predict the appearance of coronary events [59]. Previous works reported a decreased plasma myeloperoxidase level in response to continuous training in healthy animals [60, 61]. The only study on the effects of MICT and HIIT in sedentary healthy men in hypoxic conditions [62] found that only HIIT could reduce plasma myeloperoxidase. Plasmatic myeloperoxidase can interact with polyunsaturated fatty acids in the cell membrane, enhancing lipid peroxidation [63]. Moreover, we found that oxLDL, a marker of lipid peroxidation and a major candidate in the pathogenesis of atherosclerosis [64], was decreased only in the MICT group. This effect could contribute to reduce the cardiovascular risk. In humans, the decrease of plasma oxLDL after exercise training programs is well documented in lean older people [65] and also in people with obesity [66]. By comparing the high and low volume of intense exercise training (90% of the maximal heart rate) in healthy overweight men (BMI: 25-30), Tjonna et al. found that oxLDL was reduced only after high-volume training [16]. This difference may suggest that the total volume of HIIT in our study was too low to affect oxLDL in obese Zucker rats.

Delwing-de Lima et al. [67] evaluated the effects of MICT and HIIT on the pro/antioxidant status of obese rats induced by a high-fat diet (HFD) and compared the responses in two different tissues which are the blood and the liver. As in our study, the authors observe tissue-specific responses to MICT and HIIT. In the blood, both training protocols prevented the increase in TBARS and protein carbonyl content induced by HFD and prevented a reduction in erythrocyte CAT activity. HIIT protocol is the sole enhancing erythrocyte SOD activity. In the liver, both training protocols prevented the increase in protein carbonyl content and only MICT prevented an alteration in CAT activity. In our study, the muscle compared to adipose tissue is the most sensitive tissue to favour the upregulation of AO enzyme activities (SOD and GPX) and decreased level of AOPP, especially after the HIIT protocol. It is well established that the muscle is the tissue that produces the most ROS during exercise [68]. Since ROS produced during exercise are known to cause an activation of MAP kinases which in turn activated the NF-*κ*B pathway and consequently the expression of important enzymes associated with defense against ROS [69], it is not surprising that the muscle is the tissue where the effects of training were more pronounced regarding AO enzyme activity leading to lower oxidative damages.

Even if we demonstrated that both HIIT and MICT improved the pro/antioxidant status in a different way according to the tissues considered, our study presents limitations. The first is the use of the gastrocnemius which has the advantage of being large and has allowed us to perform all our measurements on the same tissue but has the disadvantage of being a mixed type fiber. HIIT and MICT effects may have been different on a slow muscle like soleus, especially for MICT. Only male rodents were examined and given the antioxidant effects of oestrogen; the use of a group of females would probably have revealed gender-specific responses. We also have to acknowledge that we did not evaluate the aerobic capacity of rats. However, the speed that we used for the HIIT group is close to the maximal aerobic speed (16-24 m·min^−1^) and above the speed at the lactate threshold (12.5 m·min^−1^) measured in Zucker rats in previous studies [38, 70]. In addition, the speed used for the MICT group is below the speed at the lactate threshold (10 vs. 12.5 m·min^−1^) for Zucker rats. Finally, in the beneficial effects we observed, it is impossible to distinguish what is due to the fat mass reduction or to the antioxidant effects of chronic exercise.

## 5. Conclusions

MICT and HIIT exert beneficial, but tissue-specific, effects on the pro/antioxidant status. Both training modalities can increase SOD activity in the muscle and reduce the plasma concentration of myeloperoxidase, a major cardiovascular risk indicator in plasma. As hypothesized, HIIT was more beneficial than MICT for improving the pro/antioxidant status in the muscle, where ROS are mainly produced during exercise. Conversely, MICT was more effective in epididymal adipose tissue suggesting that the two training modalities could be combined to obtain a global response in people with obesity.

## Figures and Tables

**Table 1:** Body composition pre (0 week), middle (5 weeks) and post (10 weeks) training.

	CONT			MICT			HIIT			ANOVA
	W0	W5	W10	W0	W5	W10	W0	W5	W10	G, T, GxT
Body mass (g)	376 ± 28	494 ± 41	505 ± 49	369 ± 35	491 ± 31	526 ± 37	380 ± 22	476 ± 33	493 ± 37	G: 0.315 T: 0.000 GxT: 0.017

FM (%)	35 ± 2	41 ± 1	40 ± 2	35 ± 2	39 ± 2^†^	38 ± 2	35 ± 3	38 ± 2^§^	37 ± 1^∗^^£#^	G: 0.001 (HIIT vs. CONT)^∗^ T: 0.02 GxT: 0.034 (MICT vs. CONT)^†^ (HIIT vs. CONT)^§^ (HIIT vs. CONT)^£^ (HIIT vs. MICT)^#^

FM (g)	129 ± 19	205 ± 20	201 ± 27	129 ± 17	186 ± 15^†^	203 ± 21	131 ± 22	180 ± 11^§^	180 ± 13^∗^^£#^	G: 0.043 (HIIT vs. CONT)^∗^ T: 0.000 GxT: 0.000 (MICT vs. CONT)^†^ (HIIT vs. CONT)^§^ (HIIT vs. CONT)^£^ (HIIT vs. MICT)^#^

FFM (g)	209 ± 18	252 ± 18	262 ± 22	213 ± 18	268 ± 18	281 ± 18	213 ± 15	260 ± 22	273 ± 24	G: 0.157 T: 0.000 GxT: 0.574

Data are presented as the mean ± SD. CONT: control (no exercise); MICT: moderate-intensity continuous training; HIIT: high-intensity interval training; g: gram; FM (%): percentage of fat mass; FFM: fat-free mass; W0: week 0; W5: week 5; W10: week 10. ^∗^HIIT vs. CONT: group effect (*p* < 0.05); ^†^MICT vs. CONT: group×time interaction at W5 (*p* < 0.05); ^§^HIIT vs. CONT: group×time interaction at W5 (*p* < 0.05); ^£^HIIT vs. CONT: group×time interaction at W10 (*p* < 0.05); ^#^HIIT vs. MICT: group×time interaction at W10 (*p* < 0.05). These data are included in another article dedicated to the effects of HIIT and MICT on gut-adipose tissue cross-talk in obese Zucker rats [34].

**Table 2:** Gastrocnemius muscle pro/antioxidant status after 10 weeks of exercise training.

	CONT	MICT	HIIT	Group effect
CAT (*μ*mol·min^−1^g^−1^)	2.4 ± 0.5	2.3 ± 0.6	2.2 ± 0.4	*p* = 0.8
FRAP (*μ*mol·g^−1^)	20 ± 8	24 ± 10	18 ± 5	*p* = 0.2
NADPHox (*μ*mol·min^−1^g^−1^)	0.48 ± 0.08	0.49 ± 0.08	0.49 ± 0.13	*p* = 0.9
XO (*μ*mol·min^−1^g^−1^)	0.29 ± 0.06	0.29 ± 0.06	0.31 ± 0.07	*p* = 0.7

Data are presented as the mean ± SD. CONT: control (no exercise); MICT: moderate-intensity continuous training; HIIT: high-intensity interval training; CAT: catalase; FRAP: ferric-reducing antioxidant power; NADPHox: nicotinamide adenine dinucleotide phosphate oxidase; XO: xanthine oxidase activity.

**Table 3:** Adipose tissue pro/antioxidant status after 10 weeks of exercise training.

	CONT	MICT	HIIT	Group effect
*Subcutaneous adipose tissue*				
CAT (*μ*mol·min^−1^g^−1^)	14 ± 5	14 ± 4	11 ± 3	*p* = 0.1
FRAP (*μ*mol·g^−1^)	102 ± 28	109 ± 39	115 ± 32	*p* = 0.6
SOD (*μ*mol·min^−1^g^−1^)	38 ± 15	29 ± 23	35 ± 24	*p* = 0.6
GPx (*μ*mol·min^−1^g^−1^)	19 ± 6	16 ± 8	15 ± 6	*p* = 0.4
NADPHox (*μ*mol·min^−1^g^−1^)	0.8 ± 0.3	0.7 ± 0.2	0.7 ± 0.1	*p* = 0.4
XO (*μ*mol·min^−1^g^−1^)	1.2 ± 0.5	1.0 ± 0.4	1.0 ± 0.5	*p* = 0.7

*Epididymal adipose tissue*				
AOPP (*μ*mol·g^−1^).	133 ± 52	183 ± 66	173 ± 46	*p* = 0.08
SOD (*μ*mol·min^−1^g^−1^)	69 ± 29	108 ± 40	95 ± 45	*p* = 0.06
NADPHox (*μ*mol·min^−1^g^−1^)	3.0 ± 0.8	3.7 ± 1.6	2.8 ± 1.0	*p* = 0.2
XO (*μ*mol·min^−1^g^−1^)	1.8 ± 1.1	2.8 ± 1.5	2.2 ± 1.4	*p* = 0.3

Data are presented as the mean ± SD. CONT: control (no exercise); MICT: moderate-intensity continuous training; HIIT: high-intensity interval training; CAT: catalase activity; FRAP: ferric-reducing antioxidant power; SOD: superoxide dismutase; GPx, glutathione peroxidase; NADPHox: nicotinamide adenine dinucleotide phosphate oxidase; XO: xanthine oxidase activity; AOPP: advanced oxidation protein product.

## Data Availability

The data used to support the findings of this study are available from the corresponding author upon request.
